# Laryngo-Tracheotomy

**Published:** 1874-08

**Authors:** R. J. Prestou


### LARYNGO-TRACHEOTOMY.

### FOR THE REMOVAL OF A FOREIGN BODY.

(*Read Before the Abingdon Academy of Medicine, by R. J. Prestou, M.D.*)

Infant son of Mr. and Mrs. J. R. ——, æt., ten months, was visited September 5th 1872, in consultation with Dr. R. E. Craig. On day previous, the child was discovered by his mother filling his mouth with water-melon seed, while crawling upon the floor. She at once ran to him, and removed from his mouth a dozen or more seed, after considerable resistance—strangling and coughing on the part of the child.

This coughing continued at intervals, and during the night became severe, with much surprise. Upon my arrival, Dr. C. having used all the ordihary methods for removing obstructions from the trachea, without any relief, the parents of the child were anxious for an operation to be performed. Upon auscultation, all the signs of bronchitis were present, with an occasional valve-like sound, and irregular respiratory murmur over the chest as the foreign substance was moved in coughing. The dypnea and other symptoms having abated somewhat towards the afternoon, and believing that the foreign substance might be a piece of the pulp or rind of the melon, and would soon be dissolved, upon consultation we advised against an operation, unless the symptoms should become aggravated, in which case we were to be sent for.

*Sept. 6th 8 A. M.*—Dypnea and other symptoms having increased rapidly in severity during the night, now seemed distressing, and tracheotomy was at ouce agreed upon. The child having been anæsthetized, an incision was at once made down upon the crico-thyroid membrane. There being no hemorrhage of consequence, this membrane was divided, and also the cricoid cartilage and upper ring of trachea. The larynx was now exposed to view, and being able to discover nothing above, a small probe was passed down the trachea. This excited a cough, and a water-melon seed was thrown out at the opening, with some mucus and blood. The incision was at once closed with a few stitches and adhesive strips, and the child soon rallied from the effects of the chloroform and ether. The child was crawling about playfully that evening, and the wound closed up well in a few days.

